# Searching for differentially expressed gene combinations

**DOI:** 10.1186/gb-2005-6-10-r88

**Published:** 2005-09-19

**Authors:** Marcel Dettling, Edward Gabrielson, Giovanni Parmigiani

**Affiliations:** 1 Department of Oncology, Johns Hopkins Medical Institutions, Baltimore, MD 21205, USA; 2 Department of Pathology, Johns Hopkins Medical Institutions, Baltimore, MD 21205, USA; 3 Department of Biostatistics, Johns Hopkins Medical Institutions, Baltimore, MD 21205, USA

## Abstract

We propose 'CorScor', a novel approach for identifying gene pairs with joint differential expression. This is defined as a situation with good phenotype discrimination in the bivariate, but not in the two marginal distributions. CorScor can be used to detect phenotype-related dependencies and interactions among genes. Our easily interpretable approach is scalable to current microarray dimensions and yields promising results on several cancer-gene-expression datasets.

## Abstract 2

CorScor is a novel approach to identifying gene pairs with joint differential expression. It can be used to detect phenotype-related dependencies and interactions among genes.

## Background

Gene-expression monitoring by microarray technologies has become an important approach in biological and medical research over the past decade. A common experimental design is the comparison of two sets of samples from different phenotypes (diseases and normal tissue), with the goal of searching for genes showing differential expression. This is usually done via statistical testing procedures and, often, subsequent multiple testing corrections. Prominent examples include *t*-testing, significance analysis of microarrays [1], and empirical Bayes analysis [2]. A comprehensive review of such approaches can be found in Pan [3]. All these methods use a one-gene-at-a-time strategy, considering only the association between single genes and the phenotype.

Many approaches for classification of phenotypes using microarrays do consider multiple genes simultaneously, but they address a different question, as their goal is to produce sets of differentially expressed genes for use in class prediction [4-8]. While interesting, these approaches have the limitation that they cannot be applied comprehensively to all possible pairs, that is, there currently are no practical tools for exploring phenotype-related dependencies and interactions among all gene pairs in large datasets. In this paper we present a methodology for addressing this issue, and we show that it can find interesting biological relationships that would be missed by existing approaches.

**Figure 1:**
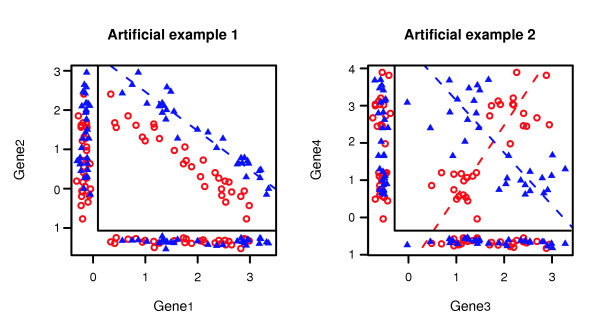
Two artificial examples of joint differential gene expression. The units of the *x*-axis and *y*-axis are gene expression; blue triangles and red circles represent samples of two different phenotypes. The inner panels reflect the joint distribution; the outer margins display the univariate marginal distributions. The dashed lines represent the first principal components, conditional on the phenotype.

We are interested in searching for two types of gene pairs, illustrated in Figure 1 by artificial examples. In the left panel, the two genes show a pronounced joint association on the phenotype: if the sum of their expression levels exceeds 3 units, we observe solely the blue-triangle phenotype. A biological mechanism leading to this phenomenon may occur when the two genes are substitutes in a molecular process that is closely linked to the phenotype. Therefore, we denote this situation as the 'substitution case'. Neither of the two genes shows a strong association with the phenotype in the univariate marginal distribution, and thus both would have been highly unlikely to appear in a gene list produced by a one-gene-at-a-time testing approach. A complementary case occurs when two genes cluster around two positively sloped axes: then the phenotype is associated with a difference in expression, a situation we refer to as the 'gap case'.

A more complex case is shown in our second artificial example, in the right panel of Figure 1. There is no obvious demarcation in space and, again, neither of the two genes carries information on its own. However, together they do. Biologically speaking, this example could reflect an 'on/off situation'. If both genes are off (expression values below 1.5 units), or both genes are on (expression value above 1.5 units), we observe the red-circle phenotype. In contrast, if only one of the genes is turned on, the blue-triangle phenotype is predominant.

Statistically, we define joint differential expression as good phenotype discrimination by the joint distribution, but not by the univariate marginal distributions of two genes. From a functional genomics perspective, such pairs could represent interesting novel biological interactions, as for example genes that are in the same pathway.

The identification of gene pairs with joint differential expression is ambitious for several reasons. First, gene pair identification is subject to the curse of dimensionality. While the usual number *p *of genes is in the tens of thousands, the number of gene pairs is *p*(*p*-1)/2, usually in the millions. Second, there are no existing and quickly computable test statistics that exactly address our notion of joint differential expression. Existing bivariate tests such as Hotelling's *T*^2 ^[9] only screen for differences in the bivariate mean vectors and will thus favor pairs that consist of genes with strong marginal effects. Third, identifying joint differential expression based on comparing predictive models for pairs and single genes is conceptually sound but is unattractive because of its prohibitive computational burden.

Here we propose a novel, efficient, and scalable approach for searching gene pairs with joint differential expression. It relies on calculating an appropriately defined test statistic from the unconditional as well as the class-conditional correlation matrices. Therefore, we call our method CorScor, as a shorthand for correlation scoring. Its biggest advantages are its straightforward interpretation and the fact that it can be calculated very quickly, which allows for an exhaustive search among the millions of pairs even in large gene-expression datasets. On the basis of several gene-expression datasets from the literature, we illustrate our method and collect empirical evidence that it yields gene pairs that have a tendency to share biological relationships.

## Results

### Data preparation

We illustrate the power and utility of our method with a comprehensive analysis of two datasets, and display the results for two further problems in the additional data files section. The first dataset discussed in detail is from a publicly available study on colon cancer by Alon *et al. *[10,11]. It originated from Affymetrix Hum6000 arrays and contains the expression values of the 2,000 genes with highest minimal intensity across 62 colon tissues, 40 of which were tumorous and 22 of which were normal. We transformed the data by a base 10 log-transformation and standardized each array to zero mean and unit variance across genes. The second is a publicly available breast cancer dataset from Hedenfalk *et al. *[12,13]. The data were obtained from Stanford-type cDNA microarrays, monitoring 2,654 genes across 22 breast cancer samples, 7 of which were found to carry germline *BRCA1 *mutations. Normalization was carried out following the approach of Yang *et al. *[14]. Our selection of data illustrates that CorScor works independently of the platform. We require accurately preprocessed expression data from *n *samples and *p *genes, stored in an (*n *× *p*) matrix denoted by (*x*_*ig*_). In what follows, we will encode the phenotype information generically as 0 and 1, and store it in the *n*-dimensional response variable *y*.

### The gap/substitution cases

Our method for revealing genes with joint differential expression relies on computing a simple score function. Given a pair consisting of genes *g *and *g'*, we determine a measure of pairwise dependence *ρ*(*g*,*g'*) among their expression vectors. Next, by restricting in turn to just the samples from each phenotype, we obtain both class-conditional measures of dependence *ρ*_0_(*g*,*g'*) and *ρ*_1_(*g*,*g'*).

For finding gene pairs that jointly discriminate the two phenotypes according to a gap or substitution mechanism as shown by the artificial example in the left panel of Figure 1, we recommend computing the scoring function

**Figure 2:**
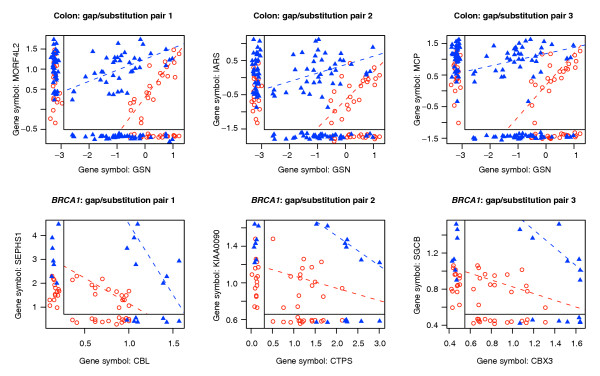
Six examples of joint differential gene expression of the gap/substitution type, obtained from the colon and *BRCA1 *datasets. The inner panels show the joint distribution; the outer margins display the univariate distributions. Blue triangles stand for cancers in colon and *BRCA1 *mutants in breast; the red circles stand for normal samples in colon and sporadic cancers in breast. The dashed lines represent the conditional first principal components.

*S*(*ρ*,*ρ*_0_,*ρ*_1_) = | *ρ*_0 _+ *ρ*_1 _- α*ρ* |     (1)

for all gene pairs (*g*,*g'*), using the Pearson correlation coefficient as dependence measure. Note that the operations in function (1) can be done for all gene pairs simultaneously by element-wise operations on three (*p *× *p*) matrices. As illustrated in Figure 2, gene pairs with high scores indeed show good joint differential expression on the colon and *BRCA1 *data, that is, accurate phenotype discrimination and comparably uninformative marginals. Some of the gene pairs we found are correlated in one group but not in the other. While this behavior does not exactly match the prototype example from Figure 1, it still fits our definition of joint differential expression. Moreover, this loss of coregulation can be a biologically relevant feature.

The rationale for the success of scoring function (1) is as follows. High conditional correlations arise if the data points within each group are tightly aligned along a straight line, which can be represented by the first principal components, shown in Figure 2 by the dashed lines. Good joint differential expression requires such tight clustering and close-to-parallel axis alignment. Hence, high conditional correlations with concordant sign, and also a shift between the alignment axes, are necessary. The bigger this shift, and thus the clearer the joint separation, the lower the unconditional correlation *ρ* gets. Hence, we diminish the sum of *ρ*_0 _and *ρ*_1 _by α*ρ*. By taking the absolute value, we achieve symmetric treatment of positively and negatively sloped alignment axes, that is, we can capture the gap and the substitution cases together. The scalar tuning parameter α governs the balance between separation and parallel alignment. We observed empirically good results with α∈ [1,2], and use α = 1.5 throughout the paper.

The first three columns in Table 1 show the values of *ρ*, *ρ*_0_, *ρ*_1_, and and the scoring function *S *for the three highest-scoring gene pairs according to the scoring function (1). As expected, the class-conditional correlations *ρ*_0 _and *ρ*_1 _tend to be high in absolute value and concordant in their signs, whereas the overall correlation is low, and sometimes even has a discordant sign.

**Figure 3:**
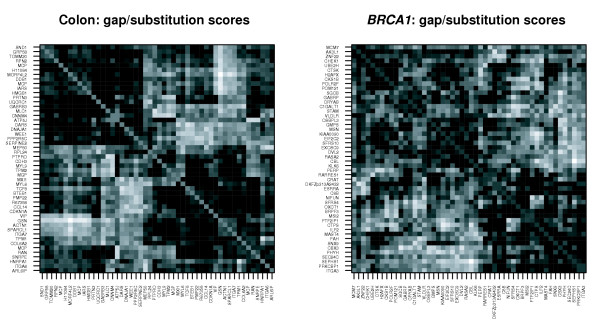
Symmetric heat map of CorScor values from Equation (1), for the colon and *BRCA1 *data. Columns and rows are rearranged according to a hierarchical clustering. Displayed are the 50 genes that are involved in the pairs with the highest scores. Black stands for low, grey for intermediate, and white for high score.

A concise visualization of the scores of gene pairs with joint differential expression is a heat map, as shown in Figure 3. We select the first 50 genes involved in the top-ranked gene pairs and color-code the score for all 50^2^/2 = 1,250 gene pairs from black (low value) through shaded grey to white (high value, excellent joint differential expression). Rows and columns of this symmetric matrix are rearranged according to a hierarchical clustering, such that genes that share common joint differential expression properties lie adjacent. We hypothesize that clustered genes may tend to share biological relationship. An exploratory analysis on the colon data supports this: the most prominent feature is a group of genes that can be found at positions 39 to 45 of the matrix. It consists of the genes with HUGO symbols *GSN*, *ACTN1*, *SPARCL1*, *ITGA7*, *TPM1*, *and COL6A2*.

Three of these six genes (*GSN*, *ACTN1*, and *SPARCL1*) share a common annotation in the Kyoto Encyclopedia of Genes and Genomes pathway database (KEGG [15]). They are all involved in the 'regulation of actin cytoskeleton'. The remaining three genes lack pathway annotation in KEGG, but an analysis of their Gene Ontology terms (GO [16]) still reveals a functional connection: *TPM1 *has the GO terms 'actin binding' and 'cytoskeleton'. *SPARCL1 *is involved in 'calcium ion binding', a term it shares with *GSN *and *ACTN1*.

The heat map of the *BRCA1 *data, shown in the right panel of Figure 3, does not show an equally pronounced block structure. The absence of KEGG annotation for a large proportion of the genes makes it challenging to carry out the same type of validation. However, consistent with the known DNA-binding function of the *BRCA1 *gene [17], many of the genes are related to binding activities. For a full overview of the genes involved in the heat maps, we refer readers to our supplementary web page [18].

Our findings on the colon data illustrate that CorScor has the potential to bring up gene pairs with a functional relationship, and that our heat maps are a helpful visualization tool for grouping and detecting the most important ones among them. The major benefit of CorScor, compared with established clustering techniques based on the expression values of single genes, is that we are able to capture genes without strong marginal effects. The genes involved in our pairs do not show pronounced fold changes across the phenotypes, but nevertheless seem to be key in molecular processes closely linked to the phenotype.

### The on/off-case

Another scenario in which joint differential expression is important is illustrated with the artificial example in the right panel of Figure 1. While the marginal distributions are not informative, the joint distribution clearly is: one phenotype is prevalent when the expression of both genes is either turned on or turned off, whereas the other phenotype is predominant when only one of the genes is expressed. An effective scoring function to capture these gene pairs is

*S*(*ρ*,*ρ*_0_,*ρ*_1_) = | *ρ*_1 _- *ρ*_0 _|,     (2)

the difference of the class-conditional dependence measures ρ_0 _and *ρ*_1_. We use Spearman's rank correlations in (2), because this prevents outlier-driven situations from appearing among the top gene pairs. Table 2 shows the values of *ρ*_0_, *ρ*_1 _and *S *for the top-scoring gene pairs in the colon and *BRCA1 *data. We observe fairly high conditional correlations here, which is partly caused by the use of Spearman's rank correlation.

**Figure 4:**
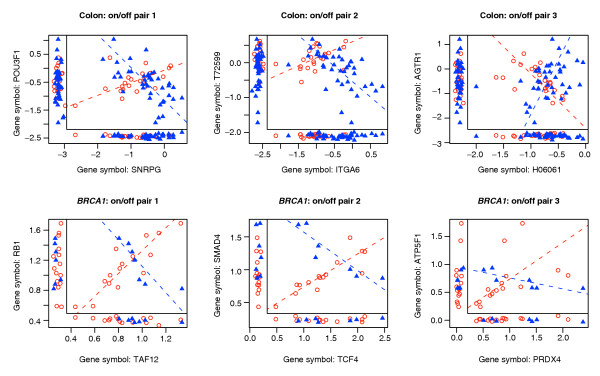
Six examples of joint differential gene expression according to the on/off-scenario, obtained from the colon and *BRCA1 *data. The inner panels show the joint distribution; the outer margins display the univariate distributions. Blue triangles stand for cancerous and *BRCA1 *mutants, the red circles for normal and *BRCA1 *wild types, respectively. The dashed lines represent the direction of the conditional first principal components.

Figure 4 shows scatterplots of the highest-scoring gene pairs on the colon and *BRCA1 *data. Joint differential expression is clearly present and an interesting biological interpretation can be derived from these scatterplots. As an example, we discuss the best-scoring gene pair from the *BRCA1 *data: for the wild-type samples (represented by red circles), there is a high positive correlation between *TAF12*, a gene that is related to transcription initiation, and *RB1*, a transcription inhibitor. For the *BRCA1 *mutant samples, the situation is reversed and the two genes show a strong negative correlation. This observation suggests a specific nuclear pathway that may be distorted as a result of *BRCA1 *mutations.

We emphasize again that because of the very different scope, such findings could not be made with one-at-a-time gene selection and/or hierarchical clustering based on gene-expression values. Again, for this on/off-scenario, the full information and annotation of the genes that are involved in the most promising gene pairs are available from our supplementary website [18].

### Permutation analysis

Next, we address the question of whether and how many gene pairs achieve promising score values by chance alone. We do this by performing permutation-based empirical Bayes analysis [2]. We generate 100 noise gene-expression datasets by scrambling the phenotype labels. We then run CorScor on each of these 100 noise datasets, obtain a vector of score values with length *p*(*p*-1)/2 and rank their values. By taking the average within rank over the 100 permutations, we obtain an estimated null distribution of CorScor values.

**Figure 5:**
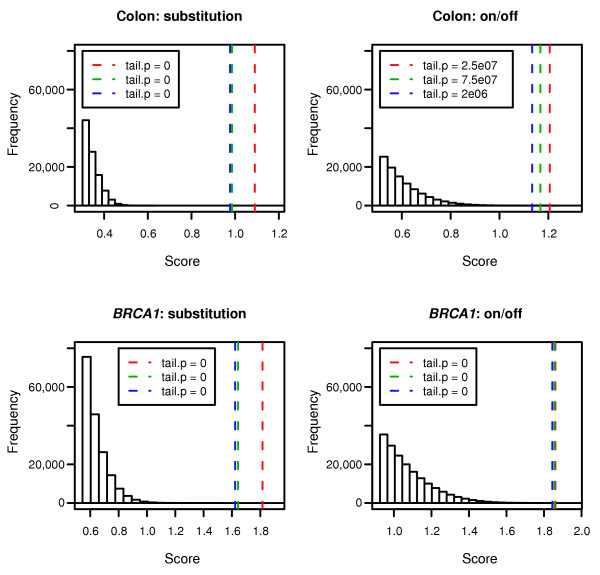
Histograms displaying the right tail of the permutation distributions of CorScor in the colon and *BRCA1 *data. The dashed vertical lines indicate the score values of the top three gene pairs from Figures 2 and 4. Also reported is the fraction of null scores (tail.p) that exceed each of observed values.

The histograms in Figure 5 display the right tail of the permutation distribution to the right of the 95% quantile. The dashed vertical lines mark the score value of the top three gene pairs (shown in Figures 2 and 4) on both the gap/substitution and the on/off situation, and for both datasets. For the top gene pairs, we also give the fraction of null scores that exceed the observed values, which is an approximation to the empirical false-discovery rate. The permutation distribution has a somewhat heavier tail and slower decay for the on/off situation. Furthermore, when comparing the colon and *BRCA1 *permutation scores, we observe that the latter have higher values. This is caused by the difference in sample size. When we arbitrarily restricted the colon dataset to the same size as the *BRCA1 *dataset, the score values were in the same range (data not shown).

Table 3 shows the number of gene pairs that exceed a given quantile of the permutation distribution, together with the ratio of observed versus expected number of gene pairs exceeding these quantiles. Again here, we observe that in the gap/substitution scenario, more gene pairs reach very high significance levels. In general, our results confirm that it is unlikely that the gene pairs we report have their joint differential expression due to chance alone.

### Comparison with predictive modeling

Next, we contrast the results of searching for jointly differentially expressed gene pairs by CorScor to an alternative search based on predictive modeling, implemented with logistic regression. This is also a novel method, although some ideas in this direction were presented in a conference talk by P. Wirapati [19]. The predictive-modeling approach is far more computer intensive and currently not applicable to arrays with tens of thousands of features. We chose the following procedure for our predictive-modeling search. In the gap/substitution situation and for each gene pair (*g*,*g'*), we fitted three logistic regression models: a model with both genes as additive inputs to capture bivariate differential expression, and two univariate models with each gene as input to capture the marginal separation. This generates conditional probability estimates *p*_*i*_(*x*_*g*_, *x*_*g'*_), *p*_*i*_(*x*_*g*_), and *p*_*i*_(*x*_*g'*_) for each observation *i*. We then compute three log-likelihoods on the basis of these probabilities,



The log-likelihood is a very natural measure for the amount of discrimination in binary problems. A gene pair with good joint differential expression reflecting a gap or substitution should show good discrimination for the bivariate model but comparably poor discrimination for the single-gene models. Hence, we can define a scoring function based on predictive modeling as



**Figure 6:**
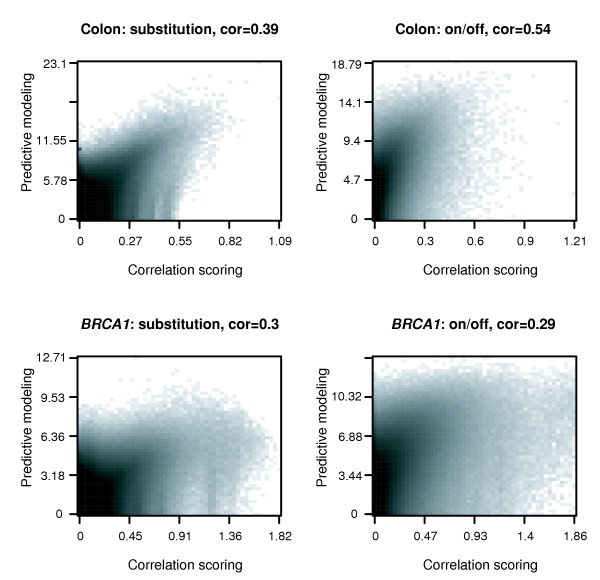
Comparison of CorScor and predictive modeling scores. Density plots for a comparison of the gap/substitution scoring function from correlation scoring defined in Equation (1) and predictive modeling (Equation (4)), as well as the on/off objective measures defined in Equations (2) and (5). Each panel is divided into a 50-×-50-cell grid. The darker the color of a cell, the more instances are therein. In the figure header, cor is the Pearson correlation coefficient between the CorScor and the respective predictive modeling scores.

The left two panels in Figure 6 show scatterplots of CorScor's outcome versus predictive-modeling scores in the gap/substitution situation. The correlation between the two measures is 0.39 for the colon data, and 0.30 for the *BRCA1 *data.

The on/off-scenario requires a different approach. For each gene pair (*g*,*g'*), we chose to measure the improvement in predictive accuracy when comparing a full two-gene interaction model versus a two-gene additive model. This requires generating conditional probability estimates *p*_*i*_(*x*_*g*_,*x*_*g'*_,*x*_*gg'*_) and *p*_*i*_(*x*_*g*_, *x*_*g'*_) using logistic regression for each observation *i*. These are then plugged into the log-likelihood from (3). From these, we can obtain a predictive-modeling-based scoring function for the on/off scenario via

*T*(*g*,*g'*) = *l*(*y*,*p*(*x*_*g*_,*x*_*g'*_,*x*_*gg'*_)) - *l*(*y*,*p*(*x*_*g*_,*x*_*g'*_))     (5)

The concordance of this measure with CorScor's output is illustrated in the right two panels of Figure 6. We observe a correlation of 0.54 in the colon data and 0.29 in the *BRCA1 *data, but many of CorScor's top-scoring gene pairs are not identified by predictive modeling.

**Figure 7:**
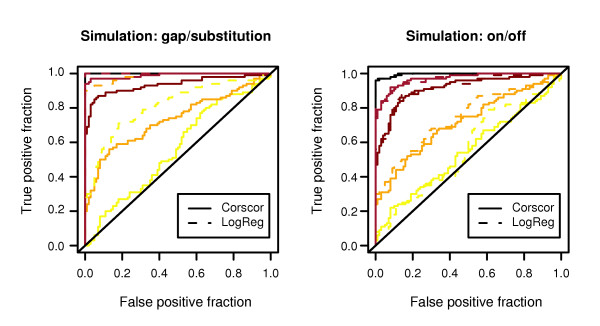
Power analysis for detecting joint differential expression. Receiver operating characteristic (ROC) curves that display the fraction of false positives, or discriminatory ability, in our simulation study to detect joint differential expression. The left panel summarizes information about the gap/substitution scenario; the right panel is about the on/off scenario. The solid lines correspond to CorScor, and the dashed ones, to logistic regression. Finally, the strength of joint differential expression was set at five different levels in our simulation experiment. The yellow lines are for the weakest amount of joint differential expression and the black lines, for the strongest amount.

For further investigation of these differences between CorScor and logistic regression, we performed a simulation study that makes it possible to judge differences in the power for detecting joint differential expression. We adopt a scenario similar to the colon data, with two phenotypes of 22 and 40 samples each. For the gap/substitution situation, the gene expressions for the two phenotypes are simulated independently according to a bivariate normal distribution with conditional correlations of 0.6. The amount of joint differential expression is controlled via a shift in the means on both axes, staggered at  standard deviations. We consider the gene pairs without mean shift (and thus with overlapping data point clouds) as the null situation without joint differential expression. The situation with  standard deviations of mean shift approximately corresponds to the amount of joint differential expression in the best gene pairs from the colon data. We generated 100 such gene pairs, determined the score values for CorScor and logistic regression, and display the ability of detecting joint differential expression with receiver operating characteristic (ROC) curves in Figure 7. We observe that logistic regression does better for the slight mean shifts, but for a moderate to large amount of joint differential expression, the two methods perform equally well.

For the on/off-scenario, the gene expressions for the two phenotypes are also simulated from independent normal distributions, but without mean shift. The amount of joint differential expression is controlled by the conditional correlations, positive for one phenotype, negative for the other. The correlation coefficients are staggered at values of  with a correlation of zero corresponding to the null situation without joint differential expression and a value of  being representative of the best pairs we see in true datasets. The right panel in Figure 7 displays the ROC curves for these simulations. We observe only slight differences between logistic regression and CorScor. Both methods show good power for detecting gene pairs with strong joint differential expression as they are found in true microarray datasets. In summary, we conclude that CorScor is as powerful at detecting relevant amounts of joint differential expression as logistic regression, but has a markedly lower computational cost.

### Software

All our computations were implemented in the statistical programming language R [20]. Via its function *cor*, it provides a very convenient and efficient routine for estimating Pearson and Spearman gene-pair correlation coefficients from an expression matrix. In the colon and *BRCA1 *data, an exhaustive search across all gene pairs with CorScor takes about 5 seconds on a 1.5 GHz Intel-Pentium-powered personal computer with 512 Mb of RAM.

All our code for identifying gene pairs with joint differential expression, as well as for their visualization by scatterplots and heat maps, is available as a documented package named *corscor*, and will be submitted to the Bioconductor project [21]. Links and updates can also be found on our supplementary website [18].

## Discussion

In a recent paper, Xiao and colleagues [22] considered multivariate searches for differentially expressed gene combinations. Their goal was to uncover subsets of predefined size *k *that are such that the multivariate distributions of expression in the two phenotypes differ. Similar ideas were used by the same group in the context of data exploration and variable selection [23,24]. The goal of their approach is to uncover sets that potentially consist of combinations of joint and marginally differentially expressed genes. This is a different goal from that considered here. For example, in Figure 4, vertically shifting all the blue points would increase multivariate difference but leave the on/off scores from Equation (2) unchanged. Here, we emphasize the search for interactions per se, because of the clearer functional genomics implications, though high multivariate distance can also be of interest. The Xiao *et al*. approach is computationally demanding because each set is evaluated by an additional cross-validation. Comprehensive exploration of all pairs is challenging and stochastic search is necessary for subsets of three or more.

In the section 'Comparison with predictive modeling', we presented an approach to screening for joint differential expression based on predictive modeling. While this shares the scope of CorScor, it is not scalable to the current dimensions of gene-expression data. A full search with predictive modeling on the colon or the *BRCA1 *data with less than 3,000 genes each requires about two weeks of central processing unit time, whereas CorScor needs only about 5 seconds. Since the number of gene pairs and thus the computing time grows quadratically with the number of genes, the analysis of a roughly quintupled Affymetrix HGU133 array with more than 12,000 genes would increase the computing time by a factor of roughly 25, making the predictive-modeling approach prohibitive for practical application. We also observed that the gene pairs found by CorScor and by the predictive-modeling approach differ. To develop a better sense of the nature of the differences, we visually compared a large number of gene pairs from the two methods (not shown). The scatterplots of the top gene pairs according to the gap/substitution predictive-modeling scoring function in Equation (4) reveal that the predictive approach is very sensitive to outliers, whereas CorScor is more robust in this regard. Additionally, the joint separation is often more pronounced with CorScor. In the on/off search, visual scatterplot inspection and examination of gene annotations favor CorScor further. The predictive-modeling objective function in Equation (5) does not seem to exactly match the scope of its correlation-based counterpart and generally did not yield any gene pairs that could serve as indicators for aberrant molecular processes.

In the on/off search, in particular, a critical difference is in the fact that pairs can show strong evidence of a reversal in the sign of the conditional correlations, while still having a substantial overlap of the two conditional distributions (see for example the top left and top right pairs in Figure 4). This can lead to a high CorScor value, but leads to only a moderate predictive score, and a small multivariate distance. These cases, however, can be highly relevant biologically, and it is important to be able to identify them. In conclusion, of the two approaches that we are proposing and investigating here, CorScor is the simpler and more efficient computationally, and it also appears to identify gene pairs that are more promising candidates for a detailed biological analysis.

Another tool for finding interactions among gene pairs is relevance networks [25]. They examine interactions among genes by thresholding covariance matrices and graphically displaying the connections among the genes whose correlations exceed the threshold. We investigated a different type of gene interactions here, namely interactions that are altered as a result of the phenotype comparison of interest. However, the type of visualization implemented in relevance networks could also be used to represent the findings of our algorithm. Moreover, our approach was illustrated here using Pearson's and Spearman's correlations, but the general idea can be extended straightforwardly to any easily computed measure of pairwise association among gene expression levels. Finally, Zhou *et al. *[26] introduced second-order expression correlations that investigate regulatory networks by exploring variation of correlations across conditions. Whereas their method focuses on concordant correlations, our approach is based on correlation differences.

## Conclusion

In summary, this paper presents a novel approach for finding gene pairs with joint differential expression. This represents a complement to the widely used one-gene-at-a-time testing approaches and the associated list-enrichment tests. The idea behind joint differential expression is to find genes that only in pairs, and not individually, discriminate two given phenotypes. These pairs make it possible to explore dependence and interaction among genes, as well as to screen for molecular processes that are linked to disease. Since the usual number of gene pairs is in the millions, there is a need for a quickly computable criterion. We propose two scoring functions, based on conditional and unconditional correlation coefficients. We show that these measures have the ability to uncover gene pairs that show promising scatterplot patterns and tend to share a biological relationship. In cancer research, a strength of CorScor lies in its potential ability to find genes that have not traditionally been involved with cancer, as they may represent new avenues for cancer cell biology and, more importantly, therapeutic intervention.

## Additional data files

The following additional data are available with the online version of this paper. To provide further evidence for the general applicability of the CorScor approach, we provide empirical results for four additional microarray problems as additional data files. Additional data file 1 is from a publicly available leukemia study by Armstrong *et al. *[27,28]. The data originated from Affymetrix HG U95A arrays and, after our normalization, feature the expression of 6,177 genes across a total of 72 samples. For the CorScor analysis, we restricted to the binary distinction of 24 samples from acute lymphoblastic leukemias (ALL) versus 28 samples from acute myeloid leukemias (AML).

Additional data file 2 is based on a dataset from a publicly available lung cancer study of Bhattacharjee *et al. *[29,30]. It also originated from Affymetrix HG U95A arrays and contains 3,171 genes after our normalization. The CorScor analysis was run on 20 carcinoid samples and 17 normal lung tissues. Additional data file 3 is a dataset from the seminal leukemia study of Golub *et al. *[31,32]. It originated from Affymetrix Hu6800 arrays. The version we used after our normalization contained the expression of 3,571 genes across a total of 72 samples, 25 of which were from patients who had acute myeloid leukemias and 47 of which were from patients with acute lymphoblastic leukemia. Additional data file 4 is our analysis of publicly available cDNA arrays from Gruvberger *et al. *[33,34]. The data in Additional data file 4 monitor 3,389 genes across 30 estrogen-receptor-negative and 28 estrogen-receptor-positive breast cancer samples.

The scatterplots in the additional data files clearly show the presence of joint differential expression for the gap/substitution situation in all four datasets. Again, our idea works here because the red and blue data points are tightly aligned along their respective principle component, yielding good conditional correlation. On the other hand, the two phenotypes are separated, resulting in a low overall correlation. Also, the scatterplots for the on/off-situation clearly show the presence of joint differential expression, and they confirm that that there are gene pairs with reverse correlation in the case and control samples.

In the tables in the additional data files, we report the results from the permutation test on each of the four datasets. They are qualitatively similar to the ones from the colon and *BRCA1 *data shown in Table 3, meaning that, again, the real gene pairs score sufficiently better than the random ones.

## Supplementary Material

Additional File 1 Data from a publicly available leukemia study by Armstrong *et al. *[27,28]. The data originated from Affymetrix HG U95A arrays and, after our normalization, feature the expression of 6,177 genes across a total of 72 samples. For the CorScor analysis, we restricted to the binary distinction of 24 samples from acute lymphoblastic leukemias (ALL) versus 28 samples from acute myeloid leukemias (AML) Click here for file

Additional File 2 A dataset from a publicly available lung cancer study of Bhattacharjee *et al. *[29,30]. It also originated from Affymetrix HG U95A arrays and contains 3,171 genes after our normalization. The CorScor analysis was run on 20 carcinoid samples and 17 normal lung tissues Click here for file

Additional File 3 A dataset from the seminal leukemia study of Golub *et al. *[31,32]. It originated from Affymetrix Hu6800 arrays. The version we used after our normalization contained the expression of 3,571 genes across a total of 72 samples, 25 of which were from patients who had acute myeloid leukemias and 47 of which were from patients with acute lymphoblastic leukemia Click here for file

Additional File 4 Our analysis of publicly available cDNA arrays from Gruvberger *et al. *[33,34]. The data monitor 3,389 genes across 30 estrogen-receptor-negative and 28 estrogen-receptor-positive breast cancer samples Click here for file

## Figures and Tables

**Table 1:** Correlation coefficients and CorScor values for the gap/substitution scenario

	Colon			*BRCA1*		
		
	Pair 1	Pair 2	Pair 3	Pair 1	Pair 2	Pair 3
*ρ*	0.19	-0.01	0.02	0.27	0.32	0.31
*ρ*_0_	0.84	0.65	0.67	-0.79	-0.20	-0.38
*ρ*_1_	0.53	0.33	0.34	-0.63	-0.96	-0.78
*S*(*ρ*,*ρ*_0_,*ρ*_1_)	1.09	0.99	0.98	1.82	1.64	1.62

Conditional and unconditional correlation coefficients, as well as the value of the scoring functions from Equation (1) with α = 1.5, for the top three gene pairs in both the colon and the *BRCA1 *data.

**Table 2:** Correlation coefficients and CorScor values for the on/off scenario

	Colon			*BRCA1*		
		
	Pair 1	Pair 2	Pair 3	Pair 1	Pair 2	Pair 3
*ρ*_0_	0.54	0.48	-0.72	0.86	0.93	0.89
*ρ*_1_	-0.67	-0.68	0.42	-1.00	-0.93	-0.95
*S*(*ρ*,*ρ*_0_,*ρ*_1_)	1.21	1.17	1.13	1.86	1.86	1.84

Conditional and unconditional correlation coefficients, as well as the value of the scoring functions from Equation (2) for the top three gene pairs in both the colon and the *BRCA1 *data.

**Table 3:** Gene pairs exceeding quantiles

	Colon: G/S		Colon: O/O		*BRCA1*: G/S		*BRCA1*: O/O	
	
Quantile	#	o/e	#	o/e	#	o/e	#	o/e
0	1,446	-	0	-	7	-	4	-
10^-6^	2,204	1.1·10^3^	1	5.0·10^-1^	45	1.3.10^1^	8	2.3·10^0^
10^-5^	5,917	3.0·10^2^	11	5.5·10^-1^	444	1.3.10^1^	69	2.0·10^0^
10^-4^	11,260	5.6·10^1^	167	8.4·10^-1^	2,473	7.0.10^0^	584	1.7·10^0^
10^-3^	22,701	1.1·10^1^	1,924	9.6·10^-1^	12488	3.6.10^0^	5,063	1.4·10^0^

The number of gene pairs (#) that exceed a given quantile of the permutation distribution in the data for colon and *BRCA1*, along with the ratio of observed versus expected (o/e) number of gene pairs exceeding this threshold. The abbreviations G/S and O/O refer to the scoring function: G/S, gap/substitution scenario; O/O, on/off scoring situation.
