# Supplementary material for: Searching for differentially expressed gene combinations
Source: Genome Biol. 2005 Sep 19;6(10):R88. doi: 10.1186/gb-2005-6-10-r88 (PMC1257471; doi:10.1186/gb-2005-6-10-r88)
Supplement: Additional File 1 — Data from a publicly available leukemia study by Armstrong et al. [27,28]. The data originated from Affymetrix HG U95A arrays and, after our normalization, feature the expression of 6,177 genes across a total of 72 samples. For the CorScor analysis, we restricted to the binary distinction of 24 samples from acute lymphoblastic leukemias (ALL) versus 28 samples from acute myeloid leukemias (AML) [file gb-2005-6-10-r88-S1.pdf]

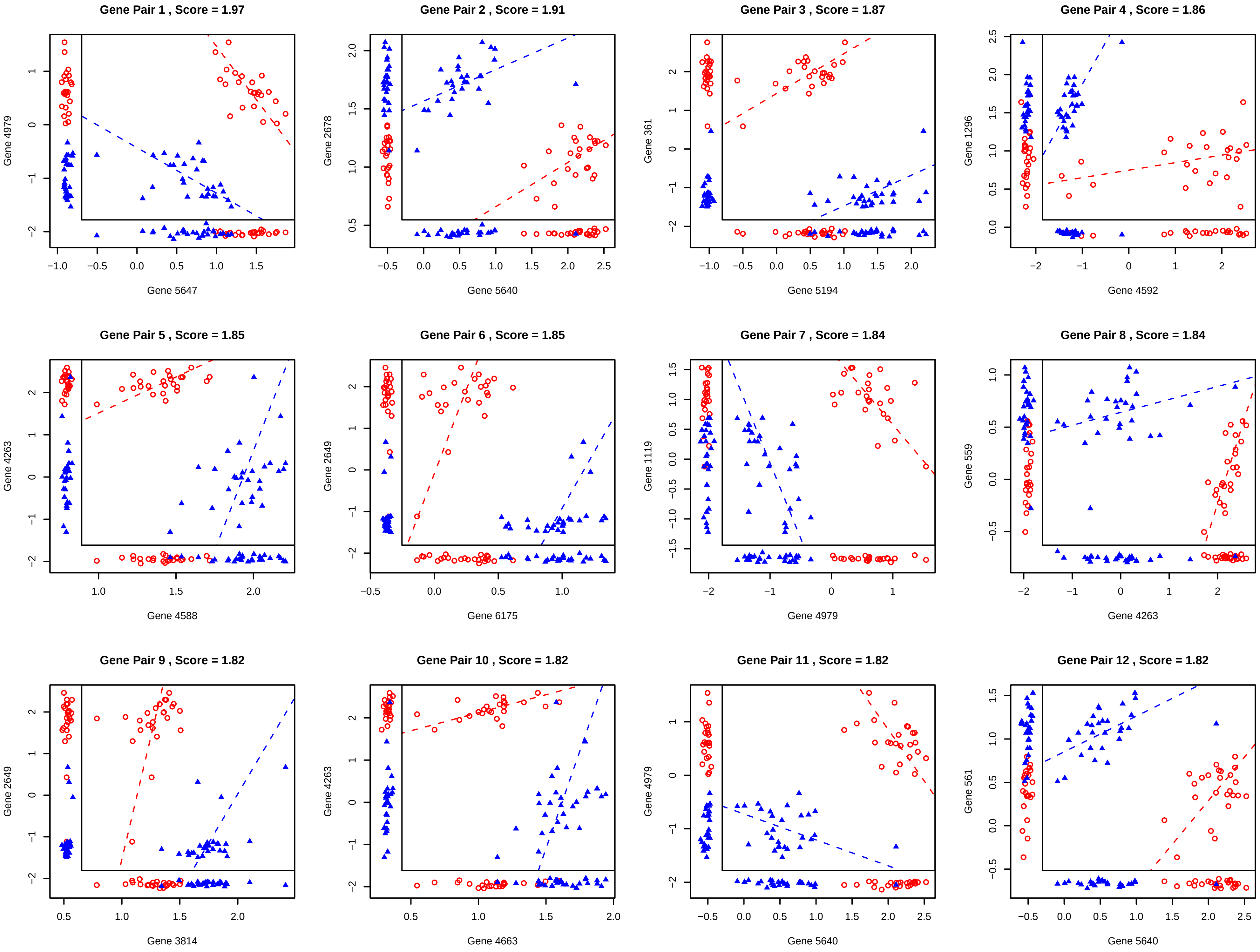
Gene Pair 1 , Score = 1.97
Gene 4979
Gene 5647
Gene Pair 2 , Score = 1.91
Gene 2678
Gene 5640
Gene Pair 3 , Score = 1.87
Gene 361
Gene 5194
Gene Pair 4 , Score = 1.86
Gene 1296
Gene 4592
Gene Pair 5 , Score = 1.85
Gene 4263
Gene 4588
Gene Pair 6 , Score = 1.85
Gene 2649
Gene 6175
Gene Pair 7 , Score = 1.84
Gene 1119
Gene 4979
Gene Pair 8 , Score = 1.84
Gene 559
Gene 4263
Gene Pair 9 , Score = 1.82
Gene 2649
Gene 3814
Gene Pair 10 , Score = 1.82
Gene 4263
Gene 4663
Gene Pair 11 , Score = 1.82
Gene 4979
Gene 5640
Gene Pair 12 , Score = 1.82
Gene 561
Gene 5640

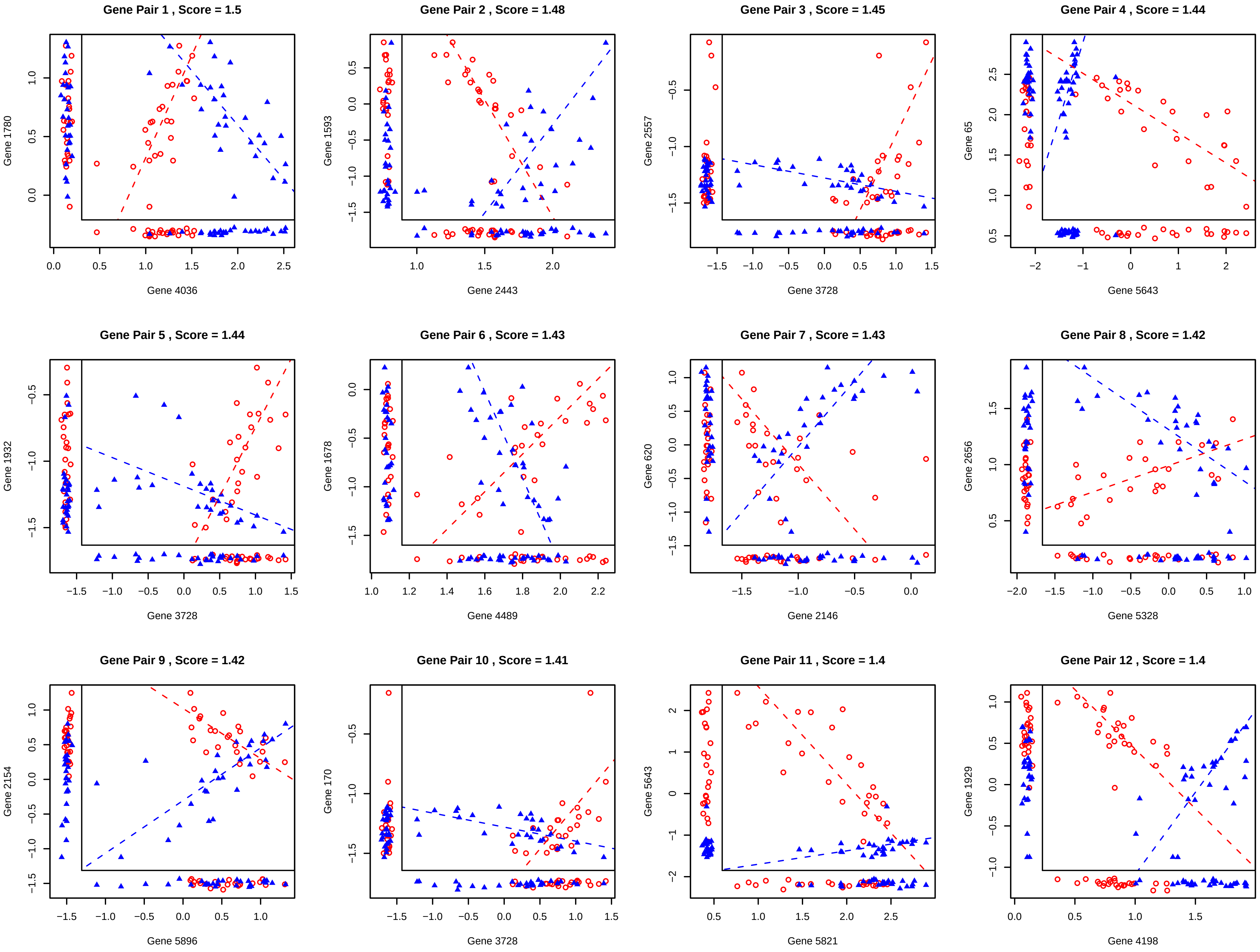
Gene Pair 1 , Score = 1.5
Gene 1780
Gene 4036
Gene Pair 2 , Score = 1.48
Gene 1593
Gene 2443
Gene Pair 3 , Score = 1.45
Gene 2557
Gene 3728
Gene Pair 4 , Score = 1.44
Gene 65
Gene 5643
Gene Pair 5 , Score = 1.44
Gene 1932
Gene 3728
Gene Pair 6 , Score = 1.43
Gene 1678
Gene 4489
Gene Pair 7 , Score = 1.43
Gene 620
Gene 2146
Gene Pair 8 , Score = 1.42
Gene 2656
Gene 5328
Gene Pair 9 , Score = 1.42
Gene 2154
Gene 5896
Gene Pair 10 , Score = 1.41
Gene 170
Gene 3728
Gene Pair 11 , Score = 1.4
Gene 5643
Gene 5821
Gene Pair 12 , Score = 1.4
Gene 1929
Gene 4198

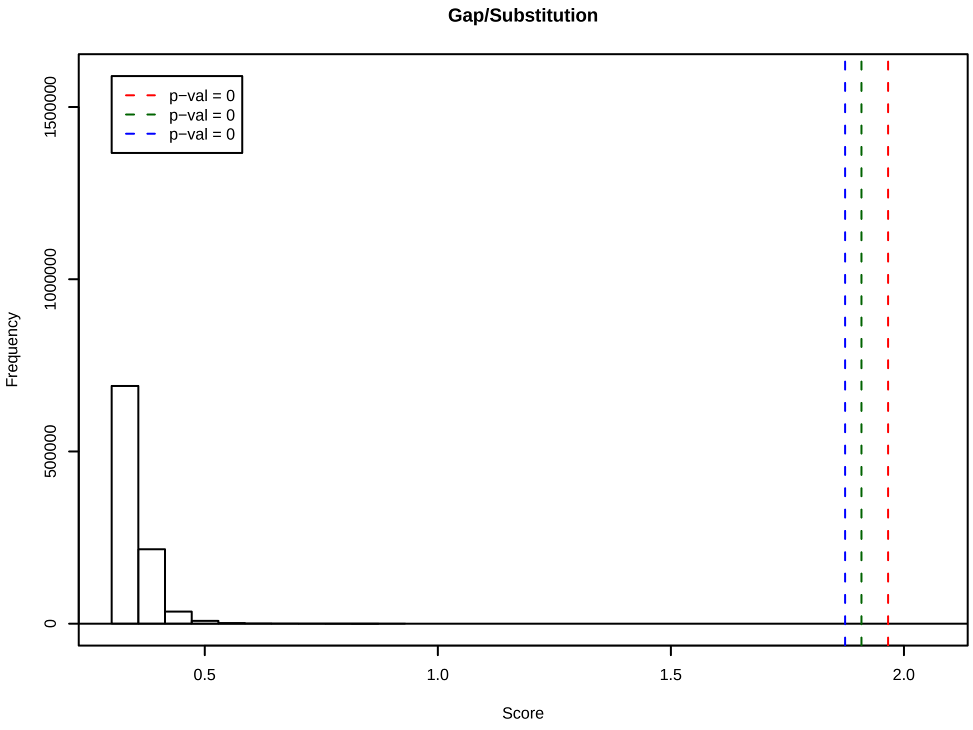

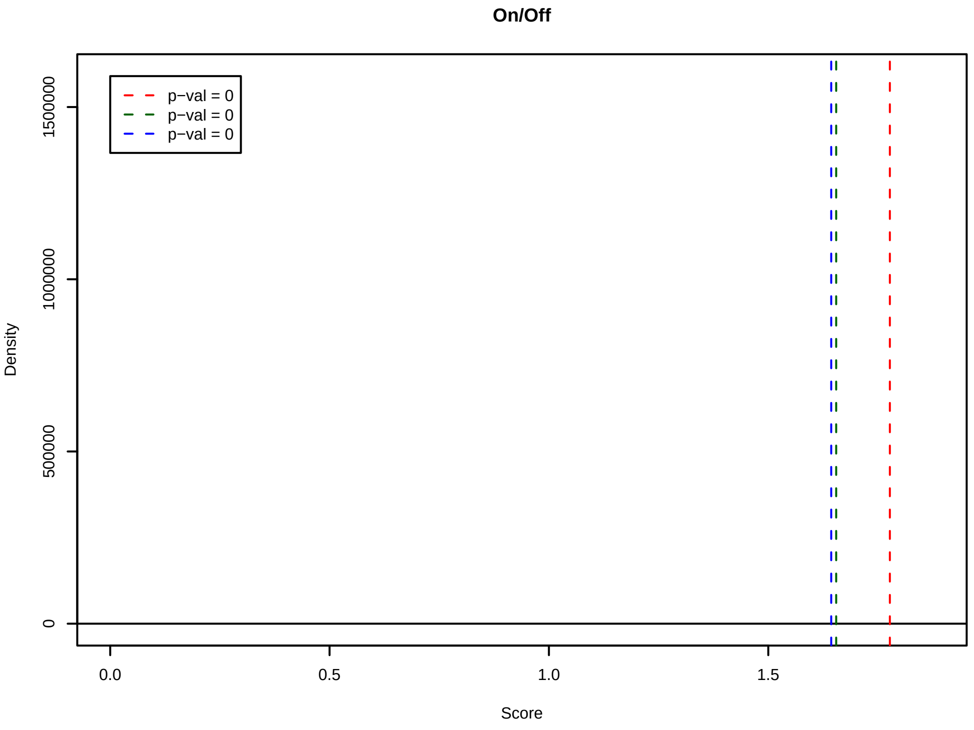

p−value	# gp exceeding	% gp exceeding	fold advantage
0e+00	138841	0.728	Inf
1e−06	209154	1.097	10965.07
1e−05	346638	1.817	1817.28
1e−04	1193706	6.258	625.81
1e−03	2889226	15.147	151.47

p−value	# gp exceeding	% gp exceeding	fold advantage
0e+00	323	0.002	Inf
1e−06	1997	0.010	104.69
1e−05	6640	0.035	34.81
1e−04	25284	0.133	13.26
1e−03	116386	0.610	6.10
