# Supplementary material for: Searching for differentially expressed gene combinations
Source: Genome Biol. 2005 Sep 19;6(10):R88. doi: 10.1186/gb-2005-6-10-r88 (PMC1257471; doi:10.1186/gb-2005-6-10-r88)
Supplement: Additional File 2 — A dataset from a publicly available lung cancer study of Bhattacharjee et al. [29,30]. It also originated from Affymetrix HG U95A arrays and contains 3,171 genes after our normalization. The CorScor analysis was run on 20 carcinoid samples and 17 normal lung tissues [file gb-2005-6-10-r88-S2.pdf]

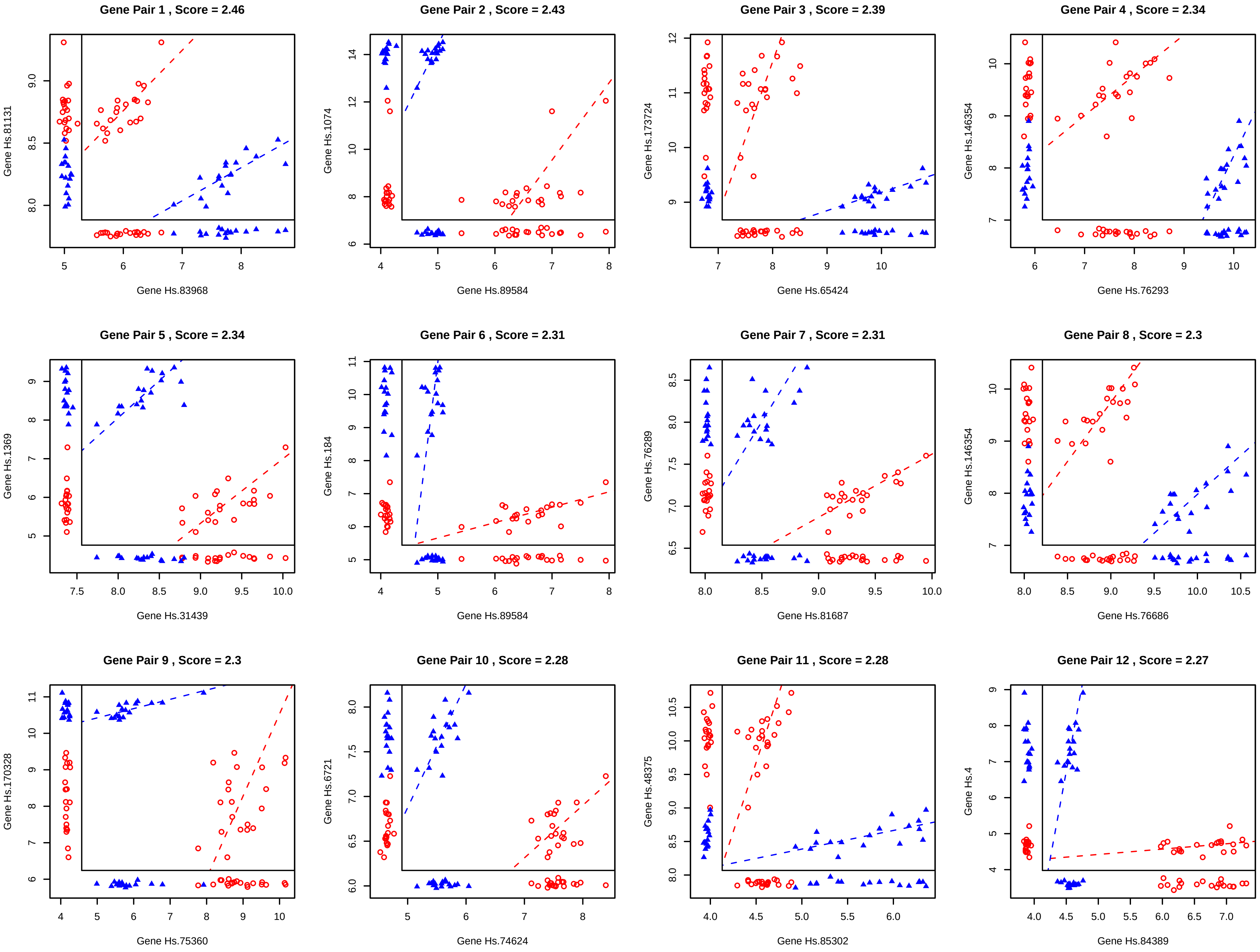
Gene Pair 1 , Score = 2.46
Gene Hs.81131
Gene Hs.83968
Gene Pair 2 , Score = 2.43
Gene Hs.1074
Gene Hs.89584
Gene Pair 3 , Score = 2.39
Gene Hs.173724
Gene Hs.65424
Gene Pair 4 , Score = 2.34
Gene Hs.146354
Gene Hs.76293
Gene Pair 5 , Score = 2.34
Gene Hs.1369
Gene Hs.31439
Gene Pair 6 , Score = 2.31
Gene Hs.184
Gene Hs.89584
Gene Pair 7 , Score = 2.31
Gene Hs.76289
Gene Hs.81687
Gene Pair 8 , Score = 2.3
Gene Hs.146354
Gene Hs.76686
Gene Pair 9 , Score = 2.3
Gene Hs.170328
Gene Hs.75360
Gene Pair 10 , Score = 2.28
Gene Hs.6721
Gene Hs.74624
Gene Pair 11 , Score = 2.28
Gene Hs.48375
Gene Hs.85302
Gene Pair 12 , Score = 2.27
Gene Hs.4
Gene Hs.84389

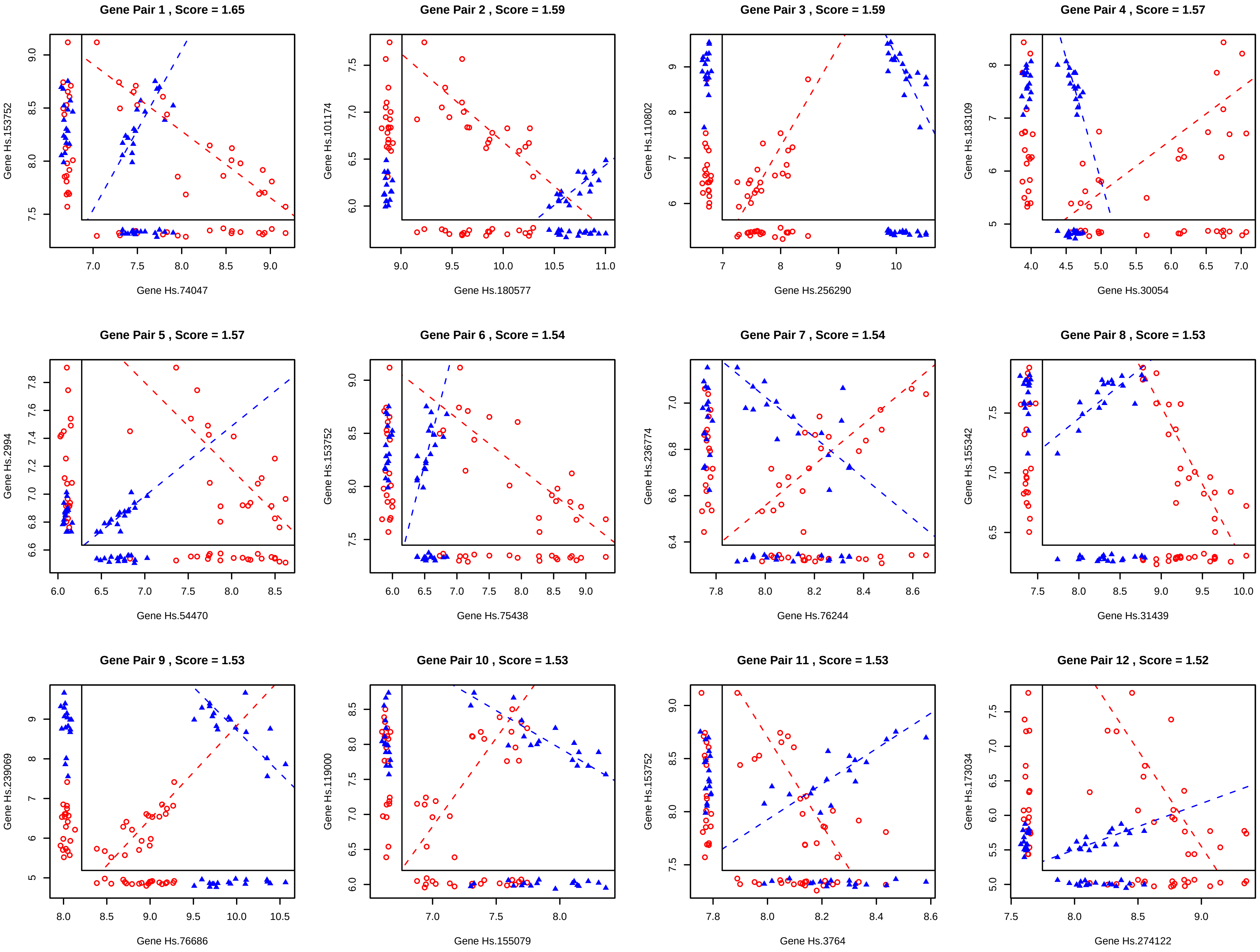

Gene Pair 1 , Score = 1.65
Gene Hs.153752
Gene Hs.74047
Gene Pair 2 , Score = 1.59
Gene Hs.101174
Gene Hs.180577
Gene Pair 3 , Score = 1.59
Gene Hs.110802
Gene Hs.256290
Gene Pair 4 , Score = 1.57
Gene Hs.183109
Gene Hs.30054
Gene Pair 5 , Score = 1.57
Gene Hs.2994
Gene Hs.54470
Gene Pair 6 , Score = 1.54
Gene Hs.153752
Gene Hs.75438
Gene Pair 7 , Score = 1.54
Gene Hs.236774
Gene Hs.76244
Gene Pair 8 , Score = 1.53
Gene Hs.155342
Gene Hs.31439
Gene Pair 9 , Score = 1.53
Gene Hs.239069
Gene Hs.76686
Gene Pair 10 , Score = 1.53
Gene Hs.119000
Gene Hs.155079
Gene Pair 11 , Score = 1.53
Gene Hs.153752
Gene Hs.3764
Gene Pair 12 , Score = 1.52
Gene Hs.173034
Gene Hs.274122

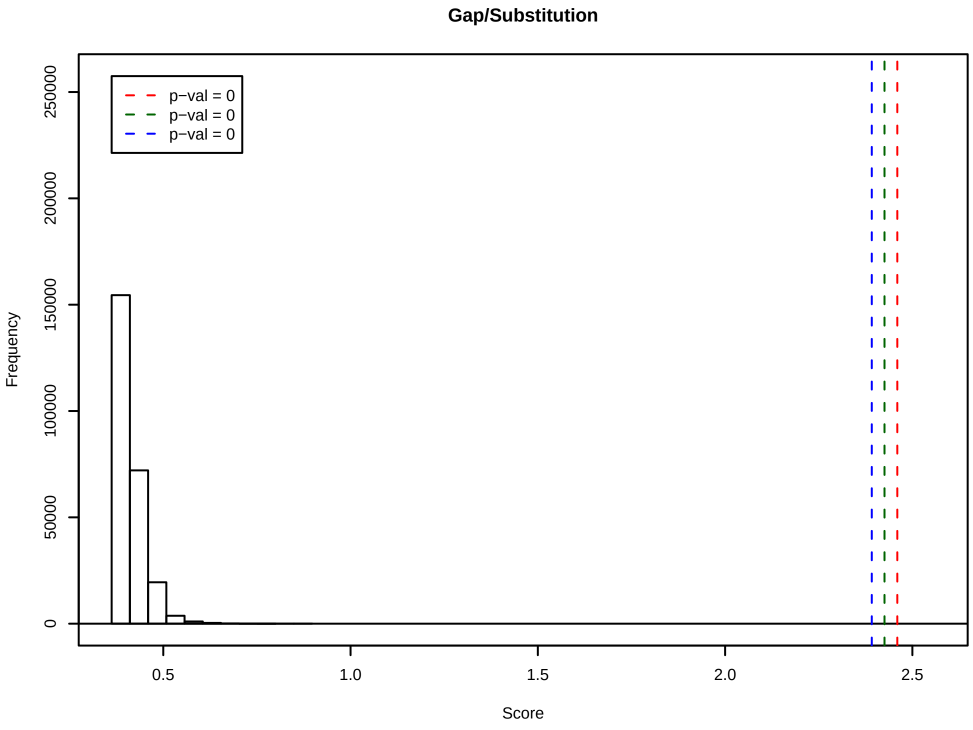

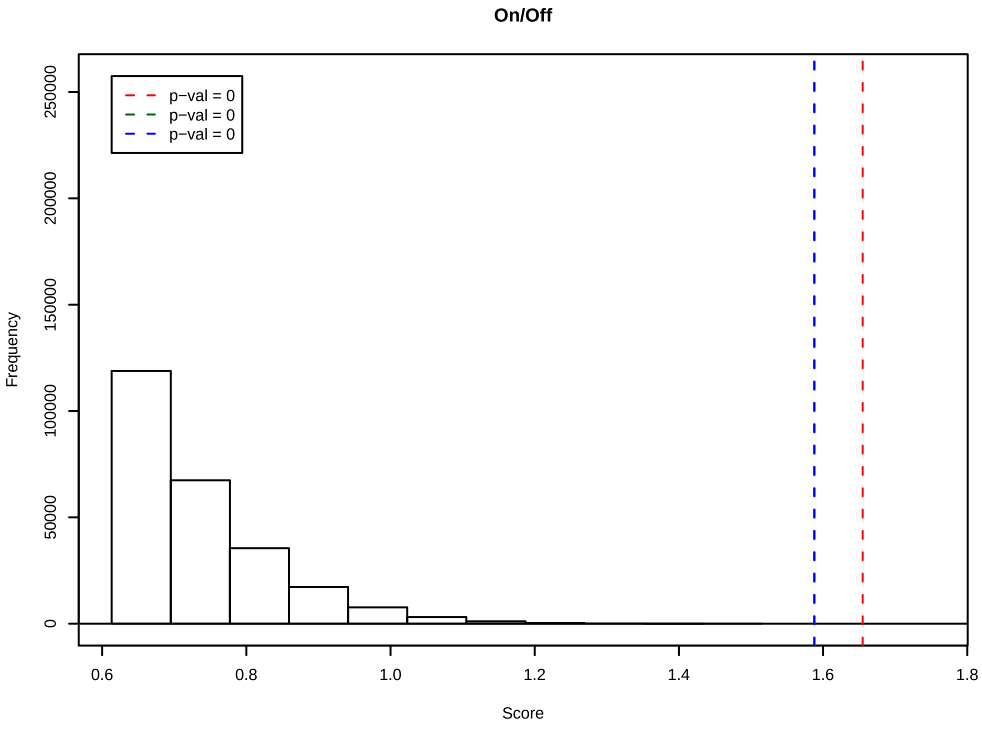

p−value	# gp exceeding	% gp exceeding	fold advantage
0e+00	351008	6.984	Inf
1e−06	498813	9.925	99245.83
1e−05	674817	13.426	13426.43
1e−04	922033	18.345	1834.51
1e−03	1243663	24.744	247.44

p−value	# gp exceeding	% gp exceeding	fold advantage
0e+00	14	0.000	Inf
1e−06	81	0.002	16.12
1e−05	481	0.010	9.57
1e−04	3045	0.061	6.06
1e−03	18805	0.374	3.74
