# Supplementary material for: Searching for differentially expressed gene combinations
Source: Genome Biol. 2005 Sep 19;6(10):R88. doi: 10.1186/gb-2005-6-10-r88 (PMC1257471; doi:10.1186/gb-2005-6-10-r88)
Supplement: Additional File 3 — A dataset from the seminal leukemia study of Golub et al. [31,32]. It originated from Affymetrix Hu6800 arrays. The version we used after our normalization contained the expression of 3,571 genes across a total of 72 samples, 25 of which were from patients who had acute myeloid leukemias and 47 of which were from patients with acute lymphoblastic leukemia [file gb-2005-6-10-r88-S3.pdf]

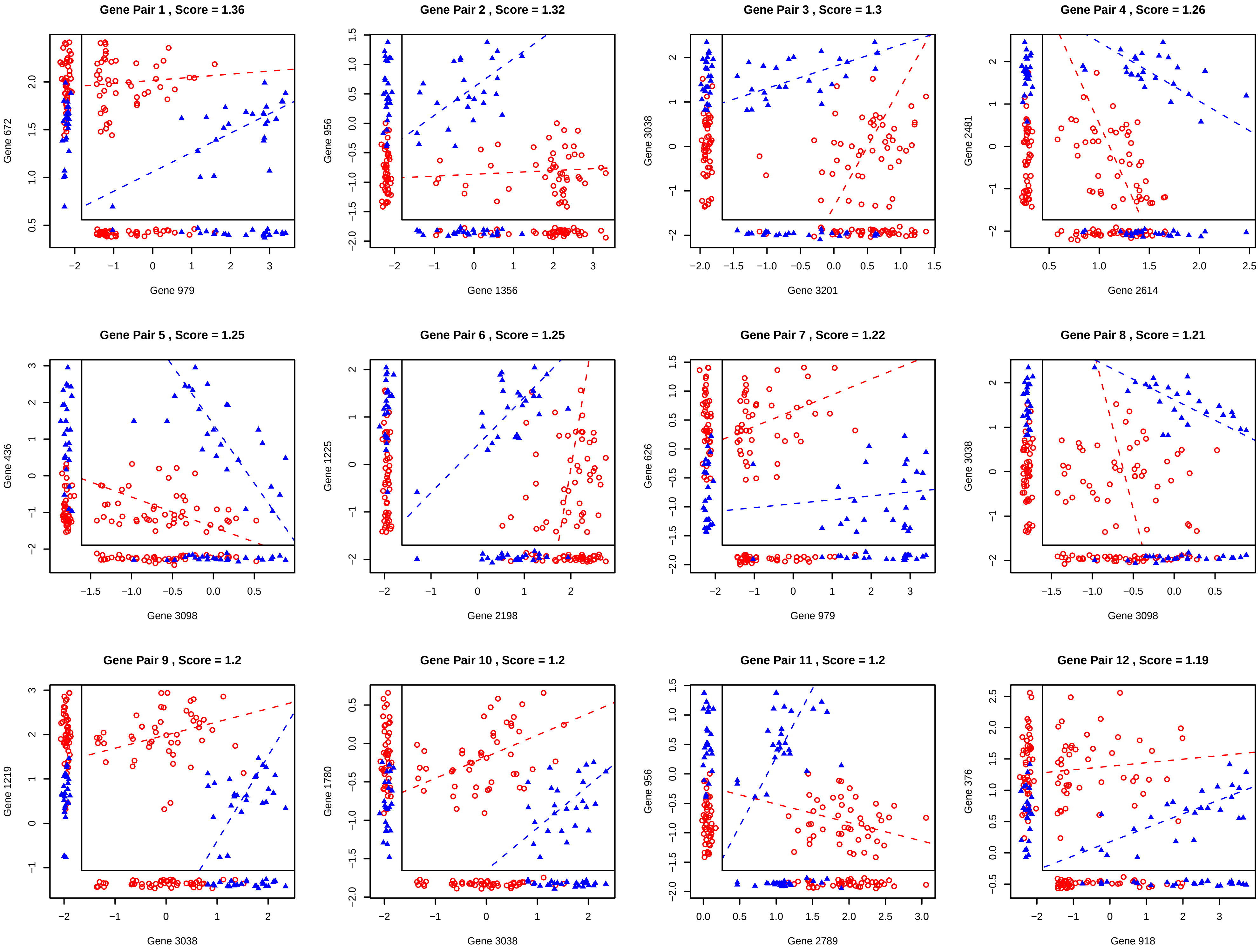
Gene Pair 1 , Score = 1.36
Gene 672
Gene 979
Gene Pair 2 , Score = 1.32
Gene 956
Gene 1356
Gene Pair 3 , Score = 1.3
Gene 3038
Gene 3201
Gene Pair 4 , Score = 1.26
Gene 2481
Gene 2614
Gene Pair 5 , Score = 1.25
Gene 436
Gene 3098
Gene Pair 6 , Score = 1.25
Gene 1225
Gene 2198
Gene Pair 7 , Score = 1.22
Gene 626
Gene 979
Gene Pair 8 , Score = 1.21
Gene 3038
Gene 3098
Gene Pair 9 , Score = 1.2
Gene 1219
Gene 3038
Gene Pair 10 , Score = 1.2
Gene 1780
Gene 3038
Gene Pair 11 , Score = 1.2
Gene 956
Gene 2789
Gene Pair 12 , Score = 1.19
Gene 376
Gene 918

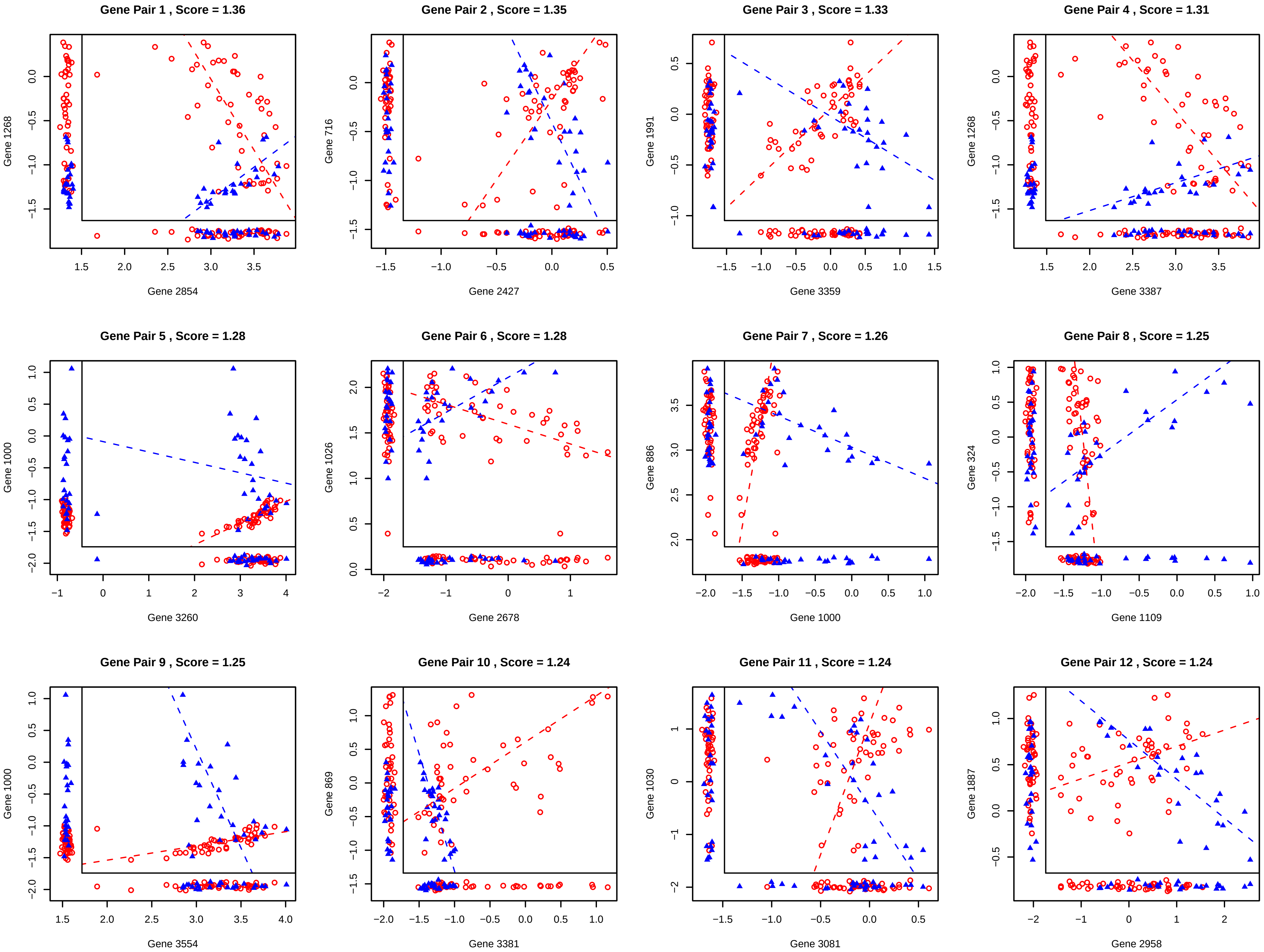
Gene Pair 1 , Score = 1.36
Gene 2854
Gene 1268
Gene Pair 2 , Score = 1.35
Gene 2427
Gene 716
Gene Pair 3 , Score = 1.33
Gene 3359
Gene 1991
Gene Pair 4 , Score = 1.31
Gene 3387
Gene 1268
Gene Pair 5 , Score = 1.28
Gene 3260
Gene 1000
Gene Pair 6 , Score = 1.28
Gene 2678
Gene 1026
Gene Pair 7 , Score = 1.26
Gene 1000
Gene 886
Gene Pair 8 , Score = 1.25
Gene 1109
Gene 324
Gene Pair 9 , Score = 1.25
Gene 3554
Gene 1000
Gene Pair 10 , Score = 1.24
Gene 3381
Gene 869
Gene Pair 11 , Score = 1.24
Gene 3081
Gene 1030
Gene Pair 12 , Score = 1.24
Gene 2958
Gene 1887

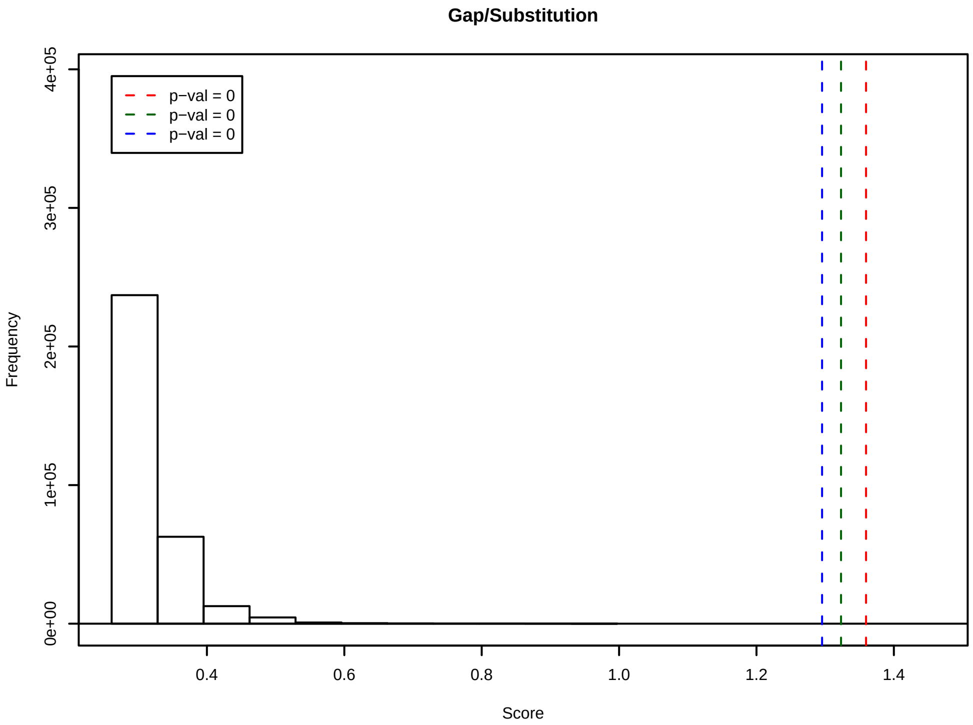

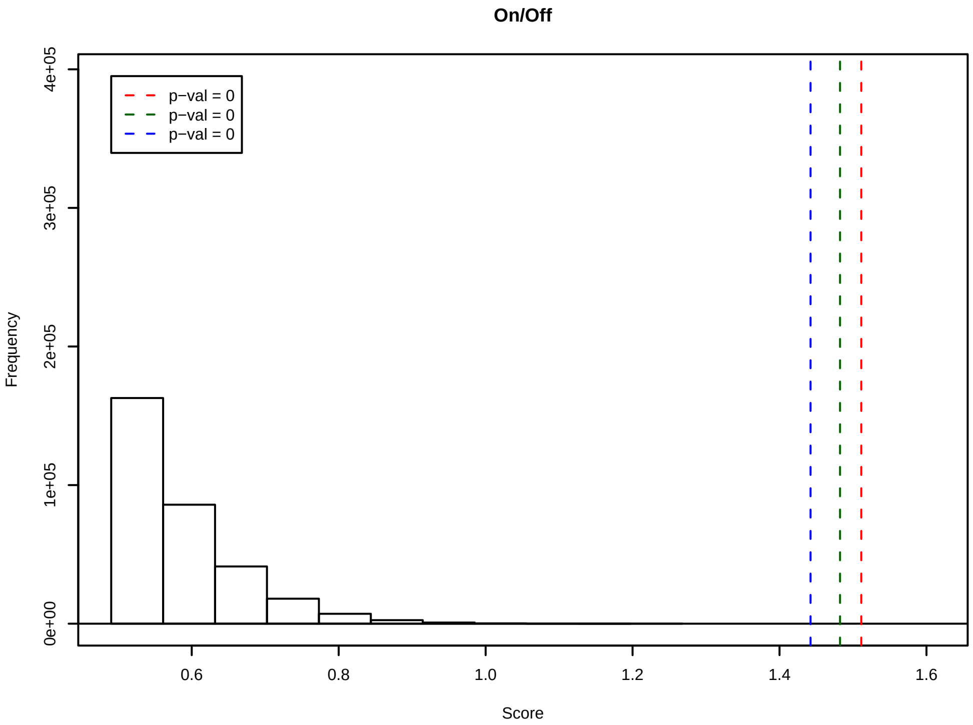

p−value	# gp exceeding	% gp exceeding	fold advantage
0e+00	194	0.003	Inf
1e−06	395	0.006	61.97
1e−05	1077	0.017	16.90
1e−04	14370	0.225	22.54
1e−03	106948	1.678	16.78

p−value	# gp exceeding	% gp exceeding	fold advantage
0e+00	136	0.002	Inf
1e−06	476	0.007	74.68
1e−05	1566	0.025	24.57
1e−04	6342	0.099	9.95
1e−03	30017	0.471	4.71
