# Supplementary material for: Searching for differentially expressed gene combinations
Source: Genome Biol. 2005 Sep 19;6(10):R88. doi: 10.1186/gb-2005-6-10-r88 (PMC1257471; doi:10.1186/gb-2005-6-10-r88)
Supplement: Additional File 4 — Our analysis of publicly available cDNA arrays from Gruvberger et al. [33,34]. The data monitor 3,389 genes across 30 estrogen-receptor-negative and 28 estrogen-receptor-positive breast cancer samples [file gb-2005-6-10-r88-S4.pdf]

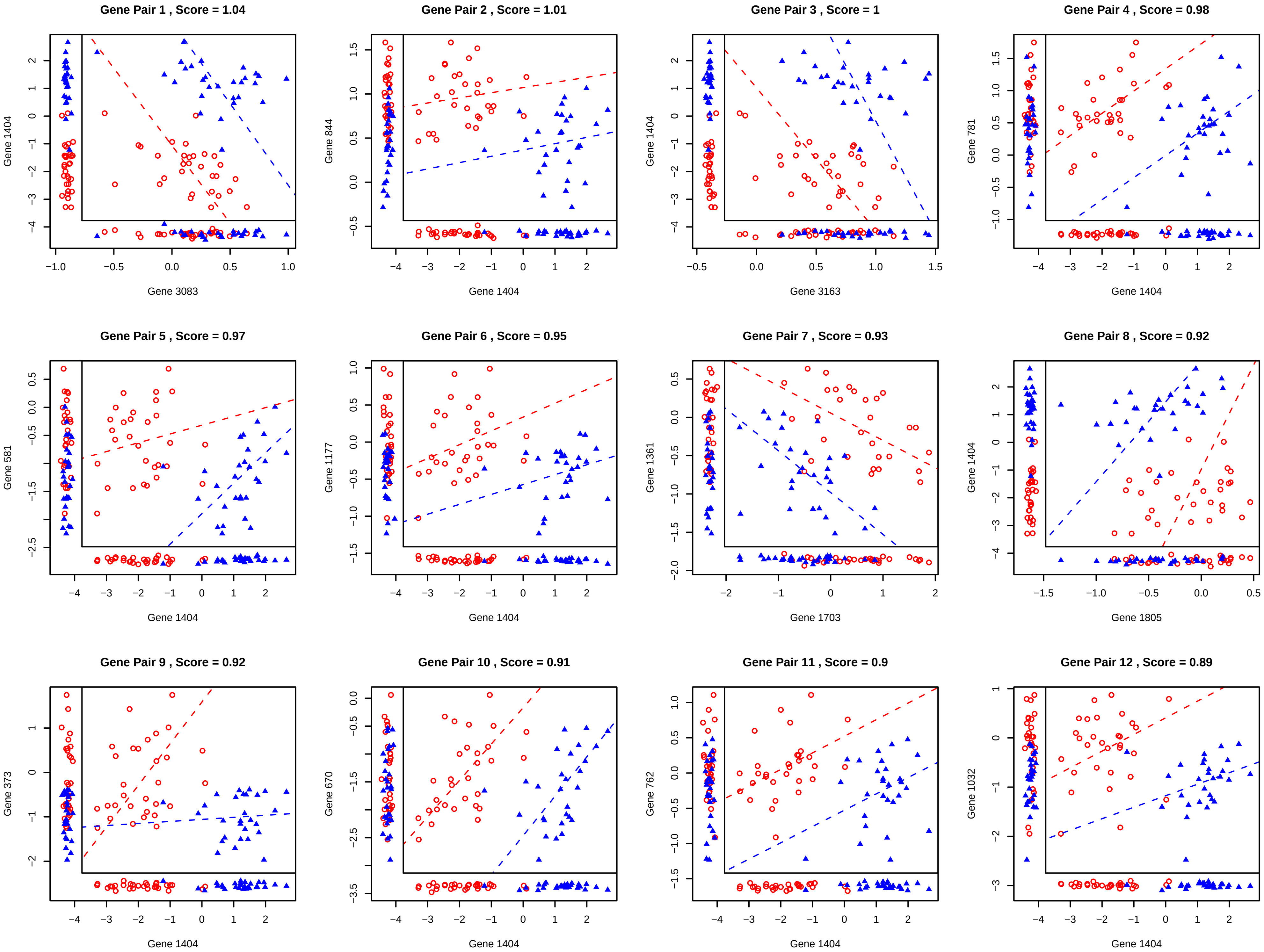
Gene Pair 1 , Score = 1.04
Gene 1404
Gene 3083
Gene Pair 2 , Score = 1.01
Gene 844
Gene 1404
Gene Pair 3 , Score = 1
Gene 1404
Gene 3163
Gene Pair 4 , Score = 0.98
Gene 781
Gene 1404
Gene Pair 5 , Score = 0.97
Gene 581
Gene 1404
Gene Pair 6 , Score = 0.95
Gene 1177
Gene 1404
Gene Pair 7 , Score = 0.93
Gene 1361
Gene 1703
Gene Pair 8 , Score = 0.92
Gene 1404
Gene 1805
Gene Pair 9 , Score = 0.92
Gene 373
Gene 1404
Gene Pair 10 , Score = 0.91
Gene 670
Gene 1404
Gene Pair 11 , Score = 0.9
Gene 762
Gene 1404
Gene Pair 12 , Score = 0.89
Gene 1032
Gene 1404

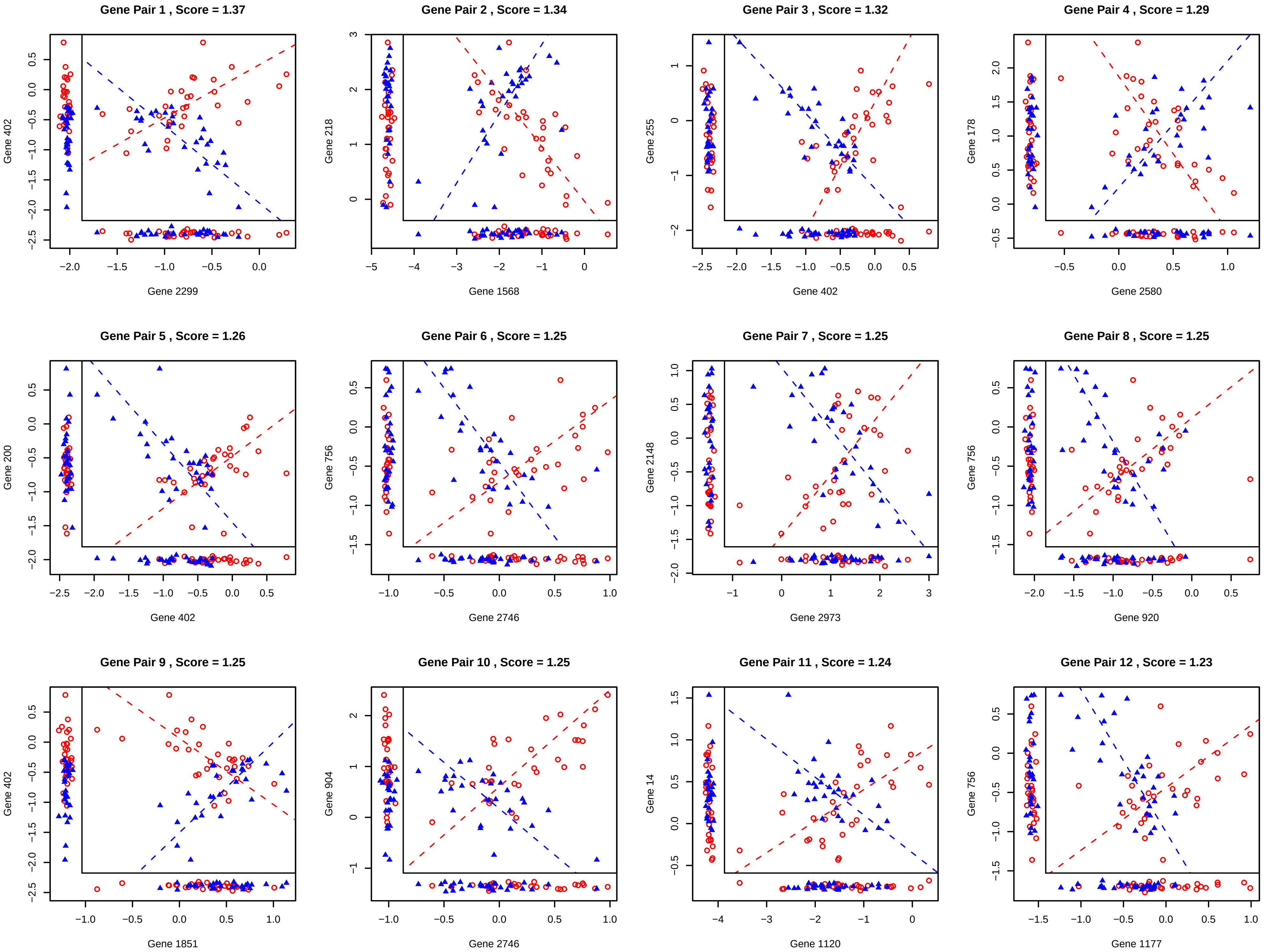

Gene Pair 1 , Score = 1.37
Gene 2299
Gene 402
Gene Pair 2 , Score = 1.34
Gene 1568
Gene 218
Gene Pair 3 , Score = 1.32
Gene 402
Gene 255
Gene Pair 4 , Score = 1.29
Gene 2580
Gene 178
Gene Pair 5 , Score = 1.26
Gene 402
Gene 200
Gene Pair 6 , Score = 1.25
Gene 2746
Gene 756
Gene Pair 7 , Score = 1.25
Gene 2973
Gene 2148
Gene Pair 8 , Score = 1.25
Gene 920
Gene 756
Gene Pair 9 , Score = 1.25
Gene 1851
Gene 402
Gene Pair 10 , Score = 1.25
Gene 2746
Gene 904
Gene Pair 11 , Score = 1.24
Gene 1120
Gene 14
Gene Pair 12 , Score = 1.23
Gene 1177
Gene 756

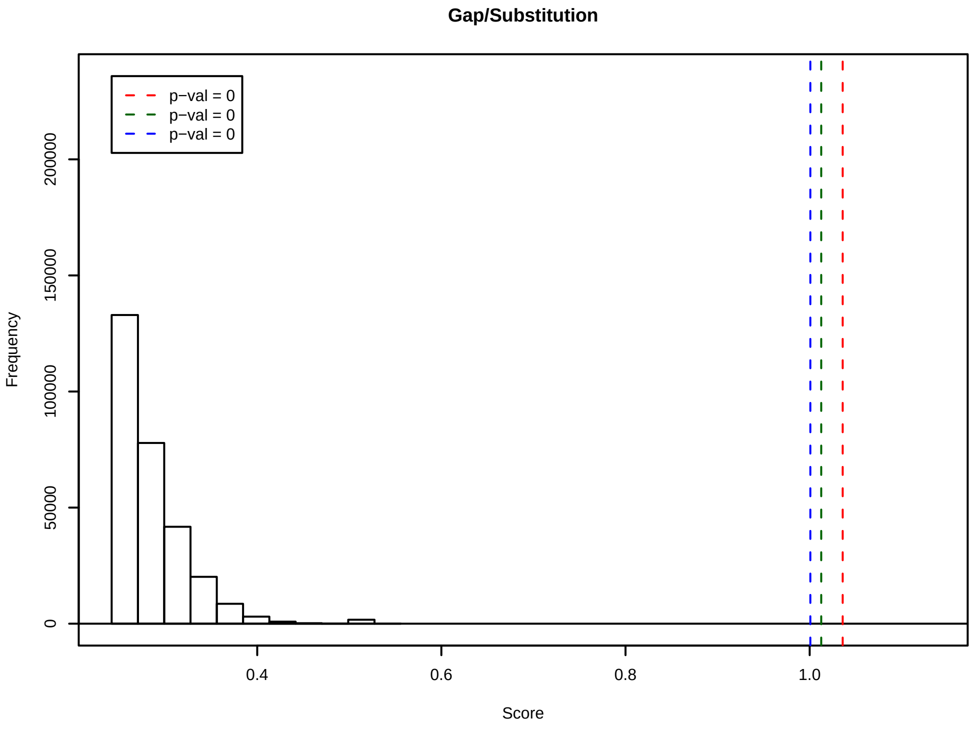

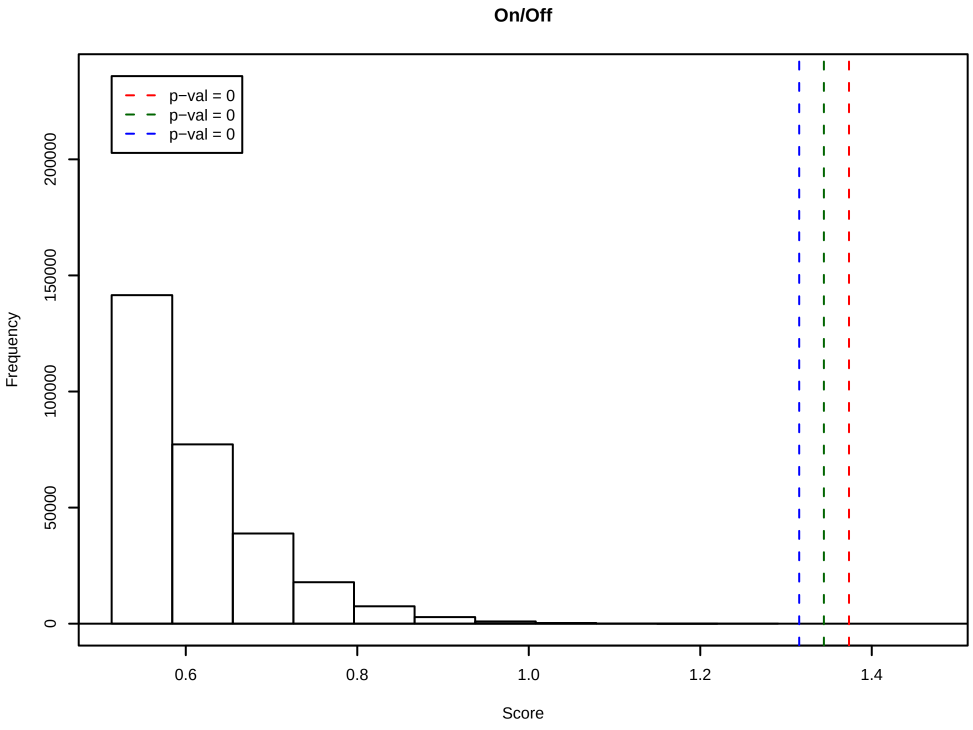

p−value	# gp exceeding	% gp exceeding	fold advantage
0e+00	3107	0.054	Inf
1e−06	5841	0.102	1017.42
1e−05	7191	0.125	125.26
1e−04	7293	0.127	12.70
1e−03	49557	0.863	8.63

p−value	# gp exceeding	% gp exceeding	fold advantage
0e+00	4	0.000	Inf
1e−06	21	0.000	3.66
1e−05	161	0.003	2.80
1e−04	1219	0.021	2.12
1e−03	9041	0.157	1.57
